# The Outcome of Extremity Soft Tissue Sarcomas in Terms of Resection Margins: A Study From a Cancer Dedicated Center

**DOI:** 10.7759/cureus.26086

**Published:** 2022-06-19

**Authors:** Muhammad bilal Shafiq, Ilyas Rafi, Ahmed Shoaib, Sajid Ali, Faizan Iqbal, Tariq Latif, Usman Mushtaq

**Affiliations:** 1 Surgical Oncology, Shaukat Khanum Memorial Cancer Hospital and Research Centre, Lahore, PAK; 2 Orthopaedic Surgery, Shaukat Khanum Memorial Cancer Hospital and Research Centre, Lahore, PAK; 3 Pediatric Surgery, Shaukat Khanum Memorial Cancer Hospital and Research Centre, Lahore, PAK; 4 Orthopedic Surgery, Baqai Medical University, Karachi, PAK; 5 Surgical Oncology, Shaukat Khanum Memorial Cancer Hospital and Research Centre, lahore, PAK

**Keywords:** outcome, recurrence, extremity, sarcoma, soft tissue

## Abstract

Introduction

Extremity soft-tissue sarcomas are uncommon malignancies of mesenchymal tissue, it accounts for <1 % of cancers and has a high recurrence rate with positive resection margins and unplanned excision. This study aims to determine the influence of unplanned excision and resection margins on local recurrence, metastasis, and overall survival in soft tissue sarcoma of the extremities.

Methods

A retrospective review was conducted from January 2005 to December 2015 on all the patients with soft tissue sarcoma of the extremities. Age, sex, histopathology, site, tumor grade, biopsy type, recurrence, metastasis, and end outcome were analyzed. Kaplan-Meir curves were used for Survival analysis, and log-rank or the Cox proportional-hazards regression model was used for Significance analysis. The data were entered into SPSS version 20, and Statistical significance was set at a p-value ≤0.05.

Results

One hundred forty-five patients with soft tissue sarcoma of extremities were managed with a mean follow-up of 76.3+/-6.7 months. Undifferentiated pleomorphic sarcoma 47 (32.4%) was the most common pathology found in this cohort, followed by Synovial sarcoma 34 (23.4%) and Liposarcoma 19 (13.1%). The most common site of occurrence was lower extremity 102 (70.3%). All the patients had residual disease after unplanned excisions; 107 underwent R0 resection, while 38 underwent R1 resection. Five-year overall survival was 70.2 & 71.1 % for R1 & R0 resections, respectively, and 71.3% for excisional and 74.2% for incisional biopsy. The tumor grade significantly influences overall survival, while other variables were not found to affect Recurrence-free survival and metastasis-free survival.

Conclusion

The data indicates that the high-grade tumor has a negative influence on overall survival, while resection margins width and unplanned excision have no significant effect on local recurrence, Metastasis free survival, and overall survival; however, before excision, adequate planning and awareness among general surgeons is necessary to improve the surgical morbidity and financial burden over the health care facilities.

## Introduction

Soft-tissue sarcomas are rare malignant tumors arising from mesenchymal tissue. These account for <1 % of all cancers [1]. In most cases (60%), the extremity is involved, and the thigh is the most common site. Soft tissue sarcomas are divided into approximately 100 histologic subtypes based on their resemblance to mature tissue. They differ in clinical behavior, molecular features, and therapy response [2,3]. An adequate and wide local excision is the standard primary treatment for soft tissue sarcoma followed by adjuvant therapy (chemoradiation) to reduce the local recurrence. Soft tissue sarcoma is difficult to treat, with approximately a third of cases recurring within two years following excision [4]. However, the prognostic effect of resection margins remains a matter of debate and the overall survival of the patient [5,6].

According to numerous studies, positive resection margins after sarcoma excision have been linked to a worse prognosis, including greater rates of local recurrence, metastasis, and lower overall survival. Kandel et al. Their meta-analysis included 33 studies; 21 studies demonstrated a negative impact of positive margins on local recurrence, except a single study did not show the same results [7]. In Another study, Bilgeri et al. concluded that a clear margin of >5 mm soft tissue seems sufficient and associated with a 05-year local recurrence-free survival of more than 80% [8]. The National Comprehensive Cancer Network (NCCN) guidelines 2020 proposed that close margins are necessary to preserve critical structures like nerves and vessels with soft tissue margins that should be <1cm [9].

Another critical issue is Soft Tissue Sarcoma's unplanned and inadequate excision, which accounts for 19% to 53% of new patients with residual or recurrent tumors seen in sarcoma centers [10-12]. The mainstay of treatment for patients who have undergone an unplanned excision is the re-resection of the tumor bed with negative margins. This practice becomes more complex with an unplanned excision of large size and high-grade tumors because of the proximity of neurovascular structures. Furthermore, positive resection margins or residual tumors after re-excision are associated with a poor prognosis, higher rates of local recurrence, disease metastases, and declined overall survival [13].

Due to insufficient health facilities and inadvertent excision, soft tissue sarcoma management is problematic in developing nations like Pakistan. One local study has shown a decrease in overall survival in the local population with closed resection margins [14]. In contrast, another study yielded no significant survival difference in planned versus unplanned excision [15]. There is a paucity of data in developing countries like Pakistan regarding the influence of resection margins on the outcome of soft tissue sarcoma excision.

Therefore, this study aims to determine the influence of unplanned excision and resection margin on local recurrence, metastasis, and overall survival in soft tissue sarcoma of the extremity. We anticipate that the resection margin will not play a role in determining local recurrence, metastasis, or the survival rate of extremity sarcoma.

## Materials and methods

This was a retrospective review of all the treated Patients with soft tissue sarcoma of extremities conducted in tertiary care hospital, in the department of Surgical Oncology from January 2005 to December 2015. Ethical review was taken from an institutional board with IRB # EX-25-01-22-02. Patients who had soft tissue sarcoma of extremity, whether primary (incisional biopsy), residual or recurrent (excisional), and underwent wide local excision (WLE) in our hospital were included. In contrast, patients with metastasis at presentation, soft tissue sarcoma involving the trunk, and those who were non-compliant about adjuvant treatment were excluded from the study.

Demographic and clinical details were retrieved from the electronic records of the patients. Variables like age, sex, histopathology, location, tumor grade, biopsy type, recurrence, metastasis, and end outcome were noted in the predesigned form. Before surgery, an MRI of the affected limb and MDT discussion were done for all patients. Metastasis was ruled out at the presentation by CT Chest. All patients (incisional or excisional biopsy) underwent WLE with adequate resection margin. A senior pathologist reviewed all tumor specimens post-operatively, and the adjuvant treatment decision was decided by a multidisciplinary team (MDT).

The tumor was graded according to FNCLCC (French Federation Nation ale des Centre's de Lutte Contre le Cancer) system. Grade 1 tumors were classified as low grade, while Grade 2 and 3 tumors were labeled high grade [16]. The margin was also defined according to the Fédération Nationale des Centres de Lutte Contre le Cancer (FNCLCC) grading system. R0 resection was defined as adequate healthy tissue around the lesion (negative microscopic margins), and R1 was the contamination of margins but an intact capsule (positive microscopic margins). Excision grossly through the tumor was classified as R2 resections. In all cases of R0 resections, patients were grouped into three categories, i.e.-margins of 1-5mm, 5-10 mm, and >10 mm.

All patients with tumor size greater than 5 cm, high grade (Grade II and III), deep to fascia, and inadequate resected margins were referred for adjuvant radiotherapy treatment as decided in MDT. All patients were closely followed for evidence of local recurrence (LR) or distant metastasis. Patients were restaged in case of disease recurrence or metastasis during follow-up (clinically or follow-up scans). The decision to resect metastatic or residual disease was individualized after discussion in the MDT. Patients have initially followed 06 monthly for recurrence for two years, then yearly afterward.

The IBM SPSS version 20 (IBM Corp., Armonk, NY, USA) was utilized for statistical analyses. Mean and standard deviations described categorical data, while frequencies and proportions described quantitative data. The patients were analyzed concerning local and distant tumor spread, with the main end-points being local recurrence-free survival (LRFS), Metastasis free survival (MFS), and Overall survival (OS) in terms of resection margins and planned excision. LRFS, MFS, and OS were defined as the time from surgery to the first occurrence of LR, metastasis, or death from any cause. Kaplan-Meir curves were used for Survival analysis, while log-rank or the Cox proportional-hazards regression model was used for significance analysis. A p-value of less than 0.05 was considered statistically significant.

## Results

Patient description

One hundred seventy-two patients underwent WLE of soft tissue sarcoma in the study period; 27 patients were lost to follow-up immediately after surgery and were excluded. The mean follow-up of patients was 76.3+/-6.7 months. Descriptive statistics of patients included in the study are shown in Table 1.

**Table 1:** Descriptive variable of patients included in the study

VARIABLE	VALUES (N=145)
SEX	
Male	83 (57.2%)
Female	62 (42.8%)
LOCATION	
Upper extremity	43 (29.7%)
Lower extremity	102 (70.3%)
BIOPSY	
Incisional	18 (12.4%)
Pin track	16 (11.0%)
Excisional	90 (62.1%)
Multiple excision	21 (14.5%)
HISTOPATHOLOGICAL DIAGNOSIS	
Undifferentiated pleomorphic sarcoma	47 (32.4%)
Synovial sarcoma	34 (23.4%)
Liposarcoma	19 (13.1%)
Leiomyosarcoma	16 (11.0%)
fibrosarcoma	23 (15.9%)
Epithelioid sarcoma	03 (2.1%)
Mayo fibrosarcoma	02 (1.4%)
Clear cell sarcoma	01 (0.7%)
GRADE OF TUMOR	
G I	09 (6.2%)
G II	81 (55.9%)
GIII	55 (37.9%)
TYPE OF RESECTION	
R0	107 (73.8%)
R1	38 (26.2%)
RESECTION MARGIN	
<1mm	38 (26.2%)
1-5mm	97 (66.9%)
5-10mm	10 (6.9%)
ADJUVANT RADIOTHERAPY	
Yes	99 (68.3%)
No	46 (31.7%)
TUMOR SIZE	
<5 cm	65 (44.8%)
>5 cm	80 (55.2%)

Incisional vs excisional biopsy

Thirty-four patients underwent wide local excision after incisional or pin biopsy. In contrast, 111 underwent unplanned excision before the presentation and had a residual disease on MRI for which re-excision was carried out. We compared the consequences of types of biopsies in terms of size of the tumor, local recurrence, metastasis, and overall survival. Tumor size was a significant variable, and large tumors (> 5 cm) were most commonly presented with incisional biopsy. Rests of the parameters were statistically insignificant, as shown in table 2.

**Table 2:** Comparison of Tumor size, Recurrence, Metastasis, and Overall survival in terms of biopsy type

VARIABLE	TOTAL	EXCISIONAL BIOPSY	INCISIONAL BIOPSY	P- VALUE
Tumor size				
<5 cm	65	55	10	0.049
>5cm	80	56	24	
Recurrence				
Yes	56	46	10	0.233
No	99	65	24	
Metastasis				
Yes	48	38	10	0.381
No	97	73	24	
Overall survival				
Alive	107	82	25	0.565
Death	38	29	9	

Resection margins (R0 vs R1)

One hundred seven patients underwent R0 resection, while 38 underwent R1 resection. The association between type of resection margin and size of the tumor, local recurrence, metastasis, and overall survival was investigated. The results are summarized in Table 3.

**Table 3:** Comparison of Tumor size, Recurrence, Metastasis, and Overall survival in terms of resection type

VARIABLE	TOTAL	R0 RESECTION	R1 RESECTION	p VALUE
Tumor size				
<5 cm	65	47	18	0.850
>5cm	80	60	20	
Recurrence				
Yes	56	38	18	0.245
No	89	69	20	
Metastasis				
Yes	48	33	15	0.422
No	97	74	23	
Overall survival				
Alive	107	78	29	0.831
Death	38	29	09	

Survival analysis

**Figure 1:**
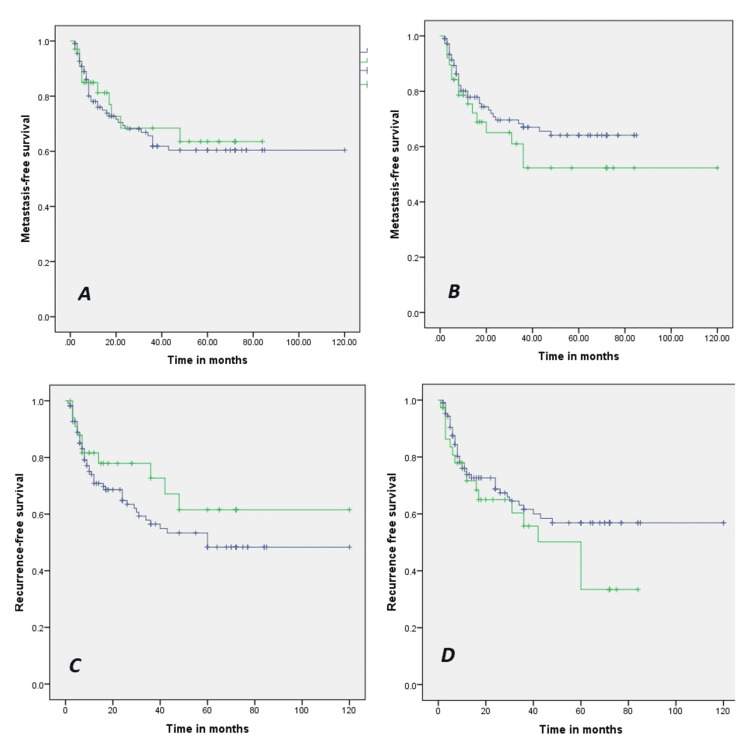
Showed survival function curves Metastasis free survival curve to biopsy showed in curve: (A) Metastasis free survival curve for resection showed in a curve; (B) Local recurrence-free survival curve to biopsy in a curve; (C) Local recurrence-free survival curve to resection showed in a curve; (D).

Five-year local recurrence-free survival was 50% for R1 resection and 56 % for R0 resection, and this difference was not statistically significant (p >0.05). Moreover, 5-year local recurrence-free survival of the patient was 48% for excisional and 62.1 % for incisional biopsy. This was also statistically insignificant (p>0.05). Five-year metastasis-free survival of the patient was 52% for R1 resection and 65 % for R0 resection, which came out to be statistically insignificant (p>0.05). Furthermore, 5-year metastasis-free survival of the patient was 61.4% for excisional biopsy and 63.9% for incisional, which was statistically insignificant (p>0.05), as shown in Fig 1.

**Figure 2:**
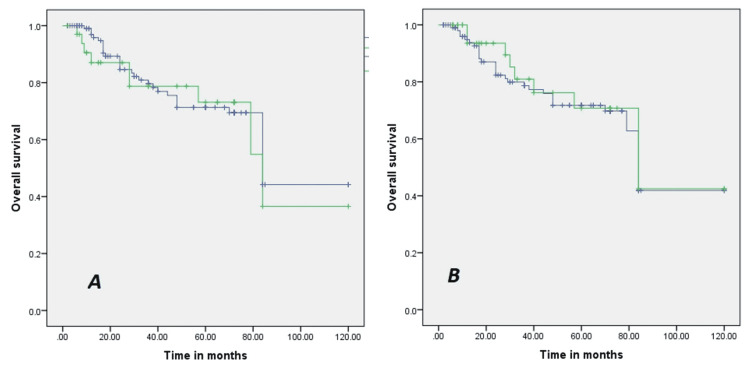
(A) The overall survival curve for a biopsy; (B) while the overall survival curve for resection

5-year overall survival of the patient was 70.2% for R1 resection and 71.1 % for R0 resection, while 5-year overall survival of the patient was 71.3% for excisional biopsy and 74.2% for incisional, which were seen as statistically insignificant (p >0.05) shown in Fig 2.

Cox regression

Cox regression on different variables affecting survival curves found that only the tumor grade can affect overall survival. In contrast, no variables were found to affect recurrence-free and metastasis-free survival, as shown in Table 4.

**Table 4:** Cox regression model for factors affecting survival

	Cox regression (b)	Standard error of the mean (SE)	Wald value	Degree of freedom (df)	Probability (p-value)	Hazard ratio Exp(B)	95.0% Confidence interval
Recurrence-free survival							
Gender	-0.133	0.281	0.224	1	0.636	0.876	0.876
Site	-0.037	0.045	0.687	1	0.407	0.964	0.964
Biopsy type	-0.462	0.352	1.725	1	0.189	0.63	0.63
Grade of tumor	0.146	0.248	0.345	1	0.557	1.157	1.157
Margin cut	-1.299	1.022	1.616	1	0.204	0.273	0.273
Type of resection	-0.995	1.087	0.838	1	0.36	0.37	0.37
Adjuvant radiotherapy	-0.173	0.341	0.258	1	0.611	0.841	0.841
Metastasis free survival							
Gender	0.084	0.301	0.078	1	0.78	1.087	0.603
Site	0.056	0.053	1.12	1	0.29	1.058	0.953
BIOPSYTYPE	-0.326	0.363	0.805	1	0.369	0.722	0.355
Grade of tumor	0.976	0.282	12.028	1	0.001	2.655	1.529
Margin cut	-0.584	0.74	0.622	1	0.43	0.558	0.131
Type of resection	-0.441	0.847	0.271	1	0.603	0.644	0.122
Adjuvant radiotherapy	0.216	0.361	0.357	1	0.55	1.241	0.611
Overall survival							
Gender	0.357	0.328	1.189	1	0.276	1.429	0.752
Site	0.026	0.064	0.164	1	0.686	1.026	0.905
Biopsy type	-0.12	0.392	0.093	1	0.76	0.887	0.412
Grade of tumor	0.978	0.314	9.685	1	0.002	2.66	1.436
Margin cut	-0.358	0.754	0.226	1	0.635	0.699	0.159
Type of resection	-0.748	0.898	0.694	1	0.405	0.473	0.081
Adjuvant radiotherapy	0.858	0.419	4.196	1	0.041	2.358	1.038

## Discussion

Soft tissue sarcomas are a heterogeneous group of rare malignant tumors prone to recurrence. In this cohort, 145 patients with extremity soft tissue sarcoma were treated, and it was observed that general surgeons inappropriately treated the majority of sarcomas at presentation under the assumption that they are benign tumors. We evaluated the effect of surgical margins and type of resection on local recurrence, metastasis, and overall survival.

Undifferentiated pleomorphic sarcoma 47 (32.4%) was the most common pathology found in this cohort, followed by Synovial sarcoma 34 (23.4%) and Liposarcoma 19 (13.1%). The commonest site of occurrence was lower extremity 102 (70.3%), which is consistent with the literature [17].

In recent times, the significance of surgical margins has been extensively studied. While tumor resections with a negative margin are considered the ultimate goal of surgical treatment for soft tissue sarcoma, the impact of the width of surgical margins and the effect of inadequate unplanned excisions on local recurrence, metastasis, and overall survival is still questionable in patients with soft tissue sarcoma [18,19]. Furthermore, we did not find any significant difference in outcomes based on margin width and unplanned excisions. In a literature review, margin status strongly correlates with local recurrence and disease-free survival [20]. However, numerous studies did not define the margin width and considered margins as either positive (microscopic residual) or negative (without residual), as the width of normal soft tissue did not affect local recurrence or survival [21-24].

Moreover, Liu et al. suggested that a 10 mm margin width is an adequate margin considering the critical threshold (20). Another study by Heslin et al. with the extremity soft tissue sarcoma found that a positive microscopic margin was significantly associated with distant metastasis and tumor mortality [25]. On the other hand, Gronchi et al., in their cohort, did not find a prognostic effect of margin status on disease-free survival, which is comparable with our findings with defined different margin statuses [22]. Likewise, Bonvalot et al. reported that the margin status did not correlate with overall survival in primary extremity soft tissue sarcoma, which is consistent with our work [26].

The use of adjuvant radiotherapy in soft tissue sarcoma is another significant factor in deciding the width of margins. Historically wider margins of greater than 5 cm were taken in the absence of radiation. However, such wide margins were not possible in most cases and resulted in limb loss. With perioperative radiation, recurrence was significantly reduced in narrow margins, making limb salvage surgery feasible in tumors surrounding vital structure [27]. In our study, nearly 70% of patients underwent postoperative radiation. However, its effect on decreasing local recurrence-free survival, metastasis-free survival, and overall survival was not statistically significant in cox regression analysis.

Another critical issue deserving attention in our population is that of unplanned excisions. Most patients in our study had an excisional biopsy (unplanned) before the presentation at our center. Surgery without planning may lead to tumor spread which is difficult to remove altogether, and limb function may be affected after re-resection due to the proximity of neurovascular structures. [28]. Most patients in this cohort had re-excision after initial treatment with wide negative margins. Moreover, several studies have demonstrated that patients undergoing re-resection have inferior outcomes compared to primary surgery [22-24]. However, in our cohort, no statistical significance was found between re-excision and outcomes such as local recurrence, metastasis, and patient survival.

In the published literature, there are variable outcomes when studying prognostic outcomes of patients who have received the unplanned resection. Lewis et al. reported that the disease-related metastasis-free survival rate was lower in patients who underwent re-resection than those who underwent planned primary surgery [29]. While Fiore et al. showed no significant differences in local relapse, metastasis, and mortality when comparing the re-excision group with the primary planned surgery group reported [30]. Furthermore, Ueda et al. reported that patients who underwent initial inadequate excision had higher local recurrence rates than those who had their planned primary surgery [31]. In Our study, local recurrence, metastasis, and mortality rates were slightly higher in patients who underwent unplanned resections, although this difference was not statistically significant.

Our study's only significant variable was the tumor grade, which significantly affected overall survival. These findings are comparable with those of Liu et al., who reported high-grade tumors with inadequate margins as having poor survival compared to low-grade tumors [19].

A strong point of our study is a large number of patients treated at tumor-dedicated centers in developing countries with an average follow-up of more than 05 years (>60 months). However, the retrospective nature is the weakness of the study. Prospective studies or multivariate analysis are required to address this controversial topic in soft tissue sarcoma treatment.

## Conclusions

The data suggest that the high-grade tumor harms overall survival, while resection margins width and unplanned excision have no statistically significant effect on local recurrence, metastasis-free survival, and overall survival; however, unplanned excisions and re excisions have a financial burden on the health care facilities and leads to surgical morbidity and difficulty to get wide local excision. Adequate planning and awareness' among surgeons are necessary to decrease unplanned excision.
